# Very low urinary marinobufagenin excretion reflects a high risk of disease progression in non-advanced CKD

**DOI:** 10.3389/fphys.2025.1527805

**Published:** 2025-02-03

**Authors:** Davide Bolignano, Marta Greco, Loredana Tripodi, Mario D’Agostino, Paola Cianfrone, Roberta Misiti, Sara Pugliese, Mariateresa Zicarelli, Michela Musolino, Daniela Patrizia Foti, Michele Andreucci, Giuseppe Coppolino

**Affiliations:** ^1^ Nephrology and Dialysis Unit, Magna-Graecia University Hospital, Catanzaro, Italy; ^2^ Department of Medical and Surgical Sciences, Magna-Graecia University, Catanzaro, Italy; ^3^ Clinical Pathology Lab, Magna-Graecia University Hospital, Catanzaro, Italy; ^4^ Department of Health Sciences, Magna-Graecia University, Catanzaro, Italy; ^5^ Department of Experimental and Clinical Medicine, Magna-Graecia University, Catanzaro, Italy

**Keywords:** chronic kidney disease, CKD progression, biomarker, marinobufagenin, renal failure

## Abstract

**Background:**

Chronic kidney disease (CKD) has now reached pandemic proportions but risk prediction towards end-stage kidney disease (ESKD) remains challenging. Kidney fibrosis is a key determinant in the transition from CKD to ESKD. In this prospective study, we investigated the prognostic significance of urinary Marinobufagenin (uMBG), a cardiotonic steroid with acknowledged pro-fibrotic activity, for stratifying the risk of CKD progression in individuals with non-advanced renal disease.

**Methods:**

After baseline uMBG measurements, 108 CKD patients (eGFR 40.54 ± 17 mL/min/1.73 m^2^) were prospectively followed up to 24 months. The study (renal) endpoint was a composite of serum creatinine doubling, eGFR decline >25% from baseline values, or ESKD requiring chronic renal replacement therapy.

**Results:**

During follow-up (mean 21 months), 32.4% of patients had progressive CKD. These individuals displayed almost halved baseline uMBG excretion as compared to others (p < 0.0001). At ROC analysis uMBG showed a remarkable diagnostic capacity on CKD progression (AUC 0.898) and patients with uMBG ≤310 pmol/L (Best ROC-derived cut-off) had a significantly faster progression to the endpoint (Log-rank 57.9; p < 0.0001). Restricted cubic splines fitting logistic and Cox-regression analyses revealed that the risk association between uMBG and CKD progression was best described by a curvilinear, inverse J-shaped trend, the highest risk associated with very low uMBG levels. This trend remained unaffected by adjustment for age, baseline eGFR, and 24 h-proteinuria.

**Conclusion:**

In individuals with non-advanced CKD, very low urinary excretion of MBG reflects a more sustained risk of CKD progression over time. Validation studies are needed to generalize these findings in larger heterogeneous cohorts.

## Introduction

Chronic kidney disease (CKD) poses a significant global health challenge, affecting over 800 million people worldwide and resulting in considerable morbidity, mortality, and cost allocation (Kovesdy, 2022; Provenzano et al., 2019a). CKD progression to end-stage kidney disease (ESKD) strengthens this burden due to the need for chronic renal replacement therapy, increased hospitalizations, and a higher risk of cardiovascular (CV) complications (Jankowski et al., 2021; Provenzano et al., 2019b). However, identifying CKD patients at risk of renal function worsening remains difficult due to the multitude of factors involved, the heterogeneous etiology, and the lack of widely generalizable prognostic models.

Progressive kidney fibrosis is at the crossroads of the CKD to ESKD transition (Reiss et al., 2024); causal factors in this process thus represent an important research target for either risk prediction or therapeutic advancement (Tummalapalli et al., 2016; Rysz et al., 2017).

Recently Marinobufagenin (MBG), an endogenous cardiotonic steroid that regulates blood pressure, volume, and sodium balance, has gained attention for its capacity to promote collagen accumulation in various tissues, including the heart, arterial vessels, and kidneys (Carullo et al., 2023).

In clinical studies, altered circulating MBG levels predict adverse cardiorenal outcomes in kidney transplant recipients (Bolignano et al., 2023b) and anticipate acute kidney injury in subjects undergoing major cardiac surgery (Bolignano et al., 2024).

Similarly, in ESKD patients on chronic dialysis, unbalanced MBG plasma levels are associated with the severity of cardiac dysfunction and may predict intradialytic complications (Bolignano et al., 2022; Bolignano et al., 2021), while in patients with arterial hypertension blood values of this hormone go along with renal damage (albuminuria) and reflect eGFR decline over time (Keppel et al., 2019).

In a recent study of patients with non-advanced CKD, we found decreased urinary Marinobufagenin (uMBG) excretion paralleling the severity of renal function impairment (Bolignano et al., 2023a).

Herein, we report results from a prospective analysis of the same cohort, which has been followed up to 24 months to assess the rate of CKD progression and the possible role of uMBG in predicting worse renal outcomes.

## Methods

### Patients’ selection

The full criteria for patient recruitment have been reported elsewhere (Bolignano et al., 2023a). In summary, CKD adults referred to the outpatient clinic at the Renato Dulbecco University Hospital in Catanzaro, Italy, were evaluated for inclusion in this observational, prospective study. Key inclusion criteria included having mild-to-moderate CKD (NFK stages 2–4; CKD-Epi eGFR between 15 and 90 mL/min/1.73 m^2^) and stable renal function, with no recorded temporary or permanent decline in eGFR (≥25%) in the 6 months prior to the study. The main exclusion criteria were the following: infections, cancer, recent cardiovascular events requiring hospitalization, active inflammatory conditions, peripheral edema, uncontrolled hypertension, severe proteinuria (>3 g/24 h), or previous kidney transplantation. The study received approval from the Local Institutional Review Board (Comitato Etico Regione Calabria-approval code 14.2023) and all participants gave written informed consent to participate.

### Clinical assessment and uMBG measurement

Before a scheduled outpatient visit, each participant underwent a comprehensive baseline assessment. Clinical, demographic, and anthropometric data were recorded using a standardized electronic case report form. Patient history and medical therapy were meticulously gathered through interviews and confirmed by reviewing patient records. Blood pressure was measured three times at rest, with the average value used for analysis. Laboratory parameters were assessed using standard clinical methods. For uMBG measurement, urine samples were collected from the second-morning urine, centrifuged at 1227 g for 15 min at 4°C, and aliquots were stored immediately at −80°C until batch analysis. uMBG levels were determined with a commercially available ELISA kit (BlueGene Biotech, Shanghai, China), following the manufacturer’s protocol. Specimens were frequently diluted to achieve the optimal concentration for the ELISA kit. Measurements were conducted in duplicate and blinded, with results expressed in pmol/L. To account for urine dilution, data analyses were repeated after normalizing uMBG levels for urinary creatinine.

### Follow-up and study endpoint

After the baseline assessment, patients were prospectively followed for up to 24 months (established end of the longitudinal phase) or until reaching the study endpoint. This latter was a composite of doubling of baseline serum creatinine, a decrease in eGFR>25% from baseline values and/or end-stage kidney disease (ESKD) requiring chronic dialysis treatment. Acute-on-chronic kidney disease, defined by a sudden transitory worsening in renal function which reverted to pre-event values, was not considered as a study endpoint, with patients experiencing this event being eventually censored at the correspondent time.

### Statistical analysis

The statistical analysis was performed using the MedCalc Statistical Software (version 14.8.1; MedCalc Software bvba), the STATA (version 18.0; StataCorp LL, Texas, USA), and the GraphPad prism (version 8.4.2, GraphPad Software, San Diego, California USA) packages. Data were presented as mean ± SD, median [IQ range], or frequency percentage as appropriate. Differences between subgroups were assessed by the unpaired t-test for normally distributed values, the Mann-Whitney U test for non-parametric values, and the chi-square followed by a Fisher’s exact test for frequency distributions. Receiver Operating Characteristics (ROC) analyses were performed to compute the areas under the curve (AUCs) and the best cut-off value (Youden Index) of uMBG in identifying patients reaching the endpoint. Kaplan-Meier curves were generated for patients with uMBG above or below the optimal, ROC-derived threshold and compared by a Log-Rank test.

Significantly different variables at baseline in individuals reaching the endpoint were tested by univariate followed by multivariate Cox regression analyses to establish time-dependent associations with the outcome. All longitudinal analyses were conducted on a time-to-first-event basis.

To explore potential non-linear relationships between uMBG and the study endpoint, crude and adjusted Cox regression hazard ratios for CKD progression were calculated across uMBG quartiles, assuming the quartile with the lowest outcome incidence as the reference category. Next, restricted cubic splines (RCS) with three knots placed at the 10th, 50th, and 90th percentile, as recommended (Harrell, 2015), were used to visually assess the shape of these non-linear associations. RCS were fitted by either logistic and Cox regression analyses, with the median or the optimal ROC-derived threshold of uMBG levels serving as the reference point for hazard ratios. The fit of the spline models was compared to standard linear models using the likelihood-ratio test, with p-values <0.05 indicating a better capacity of the cubic spline model in describing the risk distribution. Model quality was further evaluated using the Akaike information criterion (AIC) and the Bayesian information criterion (BIC). All results were considered significant at p-values ≤0.05.

## Results

### Baseline characteristics of the study population

The study population included 108 adult CKD patients with a mean age of 71.6 ± 10 years, of which 70.4% were male. The average eGFR (CKD-Epi) was 40.54 ± 17 mL/min/1.73 m^2^. Median proteinuria was 0.287 [0.124–1.035] g/24 h. The causes of CKD were diabetic kidney disease in 50% of the patients, nephroangiosclerosis in 16.7%, glomerulonephritis in 12.9%, interstitial diseases in 7.4%, and rare diseases, including ADPKD, in 3.7%. Diabetes was present in 54.6% of the patients, while cardiovascular comorbidities and hypertension affected 34% and 91.6%, respectively.

Almost all individuals were on combination antihypertensive therapy, often including a RAS blocker (82.4%). Approximately half of the patients were treated with diuretics and SGLT2 inhibitors, while the majority were on statins (64.8%) and hypouricemic agents (74.1%). Few individuals (13.9%) were on erythropoiesis-stimulating agents. Median uMBG levels in this population were 350 [250–450] pmol/L. Table 1 provides an overview of the main clinical characteristics of the study cohort.

**TABLE 1:** Main characteristics of the whole cohort and in patients stratified for the renal endpoint. Statistical differences between subgroups are highlighted in bold.

	All CKD n:108	Endpoint-no n:73	Endpoint-yes n:35	p
**Age (yrs)**	**71.6 ± 10**	**69.6 ± 9.9**	**75.7 ± 8.8**	**0.003**
Male gender (%)	70.4	74	62.9	0.26
Current smoking (%)	4.2	2.8	5.7	0.59
Diabetes (%)	54.6	58.9	45.7	0.22
History of CV disease (%)	34.2	22.8	35.6	0.34
Hypertension (%)	91.6	91.7	91.4	0.96
CKD etiology (%)				
DKD	50	53.4	42.8	0.41
Nephroangiosclerosis	16.7	16.4	17.1	0.92
GNs	12.9	13.7	11.4	0.74
Interstitial	7.4	8.2	5.7	0.64
Rare/ADPKD	3.7	4.1	2.8	0.74
Anti-hypertensive therapy				
ACEi/ARBs (%)	82.4	80.8	85.7	0.53
Beta-blockers (%)	56.5	58.9	51.4	0.53
CCBs (%)	44.4	47.9	37.1	0.31
Aldosterone antagonists (%)	5.6	6.8	2.9	0.66
Frusemide (%)	46.3	45.8	48.6	0.83
Other diuretics (%)	9.3	9.7	8.6	0.88
ESAs (%)	13.9	11.9	20.6	0.23
Statins (%)	64.8	61.6	71.4	0.31
SGLT-2i (%)	43.5	49.3	31.4	0.07
**BMI (kg/m** ^**2**^ **)**	**28.7 ± 5.3**	**29.6 ± 5.1**	**26.8 ± 5.3**	**0.03**
**Systolic BP (mmHg)**	**131.2 ± 19.2**	**122.2 ± 18.8**	**140.1 ± 17.7**	**0.03**
Diastolic BP (mmHg)	72.7 ± 10.3	72.9 ± 9.9	72 ± 10.9	0.67
**CKD-Epi eGFR (mL/min/1.73 m** ^**2**^ **)**	**40.54 ± 17**	**42.9 ± 15.1**	**32.9 ± 17.4**	**0.008**
**Serum Creatinine (mg/dL)**	**1.8 ± 0.71**	**1.52 ± 0.70**	**1.96 ± 0.71**	**0.01**
Urea (mg/dL)	76.9 ± 37.1	73.7 ± 34.2	83.1 ± 42.1	0.23
Glycemia (mg/dL)	111.2 ± 23	112.5 ± 22.9	109.2 ± 23.1	0.15
Albumin (g/dL)	4.35 ± 0.45	4.36 ± 0.46	4.34 ± 0.45	0.82
Serum Sodium (mmol/L)	140.4 ± 3.4	140.6 ± 3.1	139.9 ± 3.9	0.33
Serum Potassium (mmol/L)	4.75 ± 0.61	4.79 ± 0.57	4.66 ± 0.70	0.34
Serum Calcium (mg/dL)	9.52 ± 0.53	9.58 ± 0.54	9.41 ± 0.52	0.15
Serum Phosphate (mg/dL)	3.5 ± 0.73	3.55 ± 0.82	3.59 ± 0.55	0.81
Red blood cells (n*10^6^)	4.5 ± 0.9	4.67 ± 0.8	4.35 ± 1	0.10
**Haemoglobin (g/dL)**	**12.9 ± 1.7**	**13.2 ± 1.6**	**12.7 ± 2**	**0.02**
Platelets (n*10^3^)	223.8 ± 69	222.9 ± 71.4	225.6 ± 65.1	0.86
Total Cholesterol (mg/dL)	141 ± 32.6	138.1 ± 22.6	145.8 ± 45.2	0.06
LDL Cholesterol (mg/dL)	73 ± 30.6	72.1 ± 21.7	79.8 ± 22.6	0.07
Triglycerides (mg/dL)	103.5 [71.5–176]	113.2 [111–184.4]	99.8 [65.2–101.2]	0.06
iPTH (pg/mL)	89.2 [52.7–159.5]	90.6 [72.3–147]	96 [49–153.2]	0.21
Uric Acid (mg/dL)	5.4 ± 1.6	5.43 ± 1.73	5.27 ± 1.50	0.62
**Proteinuria (g/24h/1.73 m** ^**2**^ **)**	**0.287[0.124–1.035]**	**0.15 [0.06–0.38]**	**0.70 [0.08–0.94]**	**0.006**
Urine sodium (mg/24 h)	150.9 ± 58.9	149.9 ± 59.5	153.4 ± 59	0.33
Urine potassium (mg/24 h)	49.3 ± 16.8	51.8 ± 17.8	43.2 ± 12.7	0.07
**uMBG (pmol/L)**	**350 [250–450]**	**400 [300–965]**	**210 [90–360]**	**0.001**

Legend: ACEi, ACE-inhibitors; ADPKD, autosomal polycystic kidney disease; ARBs, angiotensin receptor blockers; BMI, body mass index; BP, blood pressure; CCBs, calcium-channel blockers; CKD, chronic kidney disease; CV, cardiovascular; DKD, diabetic kidney disease; eGFR, estimated glomerular filtration rate; ESAs, erythropoiesis-stimulating agents; GNs, glomerulonephritis; iPTH, intact parathyroid hormone; LDL, low-density lipoprotein; SGLT-2i, sodium-glucose cotransporter type-2, inhibitors; uMBG, urinary Marinobufagenin.

### Renal endpoint during the follow-up period

During a mean follow-up of 21 months (range 2–24), 35 patients (32.4%) experienced the renal endpoint. Of these, two patients needed to start chronic dialysis for severe CKD progression. No acute-on-chronic kidney disease events were reported.

At baseline, patients reaching the composite endpoint were older (p = 0.003) and had higher serum creatinine (p = 0.01), 24 h proteinuria (p = 0.006), and systolic blood pressure (p = 0.03) but lower BMI (p = 0.03), haemoglobin (p = 0.02) and eGFR (p = 0.008). Barely significant differences were present in total cholesterol (p = 0.06), LDL cholesterol (p = 0.07) and triglycerides levels (p = 0.06), urine potassium (p = 0.07), and in the percentage of SGLT2-inhibitors users (p = 0.07). No further differences emerged for the other variables recorded (p ≥ 0.10).

Differences in clinical data between patients reaching the endpoint as compared with others are summarized in Table 1.

### Diagnostic and prognostic value of uMBG

**FIGURE 1:**
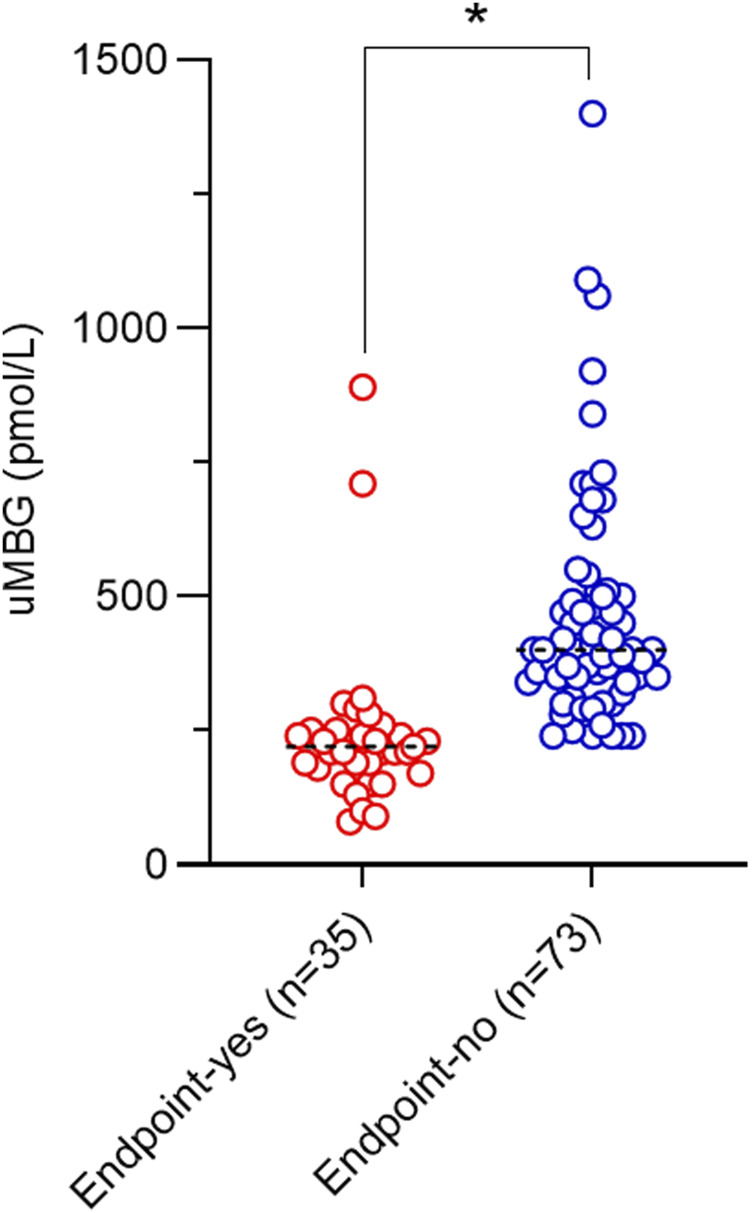
Scatterplot of baseline uMBG levels and median values (black dotted lines) in individuals stratified for renal endpoint occurrence at follow-up. *p = 0.001.

At baseline, patients experiencing the endpoint displayed significantly reduced uMBG excretion as compared to others (210 [90–360] vs400 [300–965] pmol/L; p < 0.0001; Table 1; Figure 1).

**FIGURE 2:**
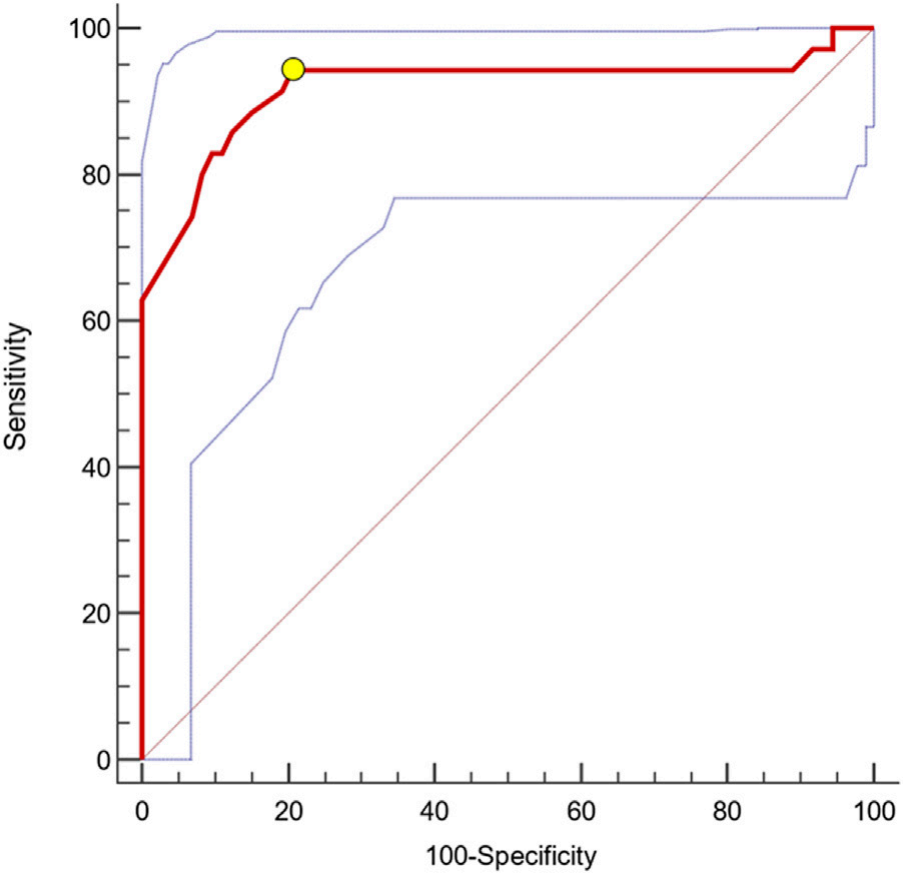
AUC (0.898) with 95%CIs (0.825–0.948; blue dotted lines) of baseline uMBG for identifying CKD patients experiencing the renal endpoint during follow-up. The best cut-off value (yellow circle) was ≤310 pmol/L (Sens. 94.3%, 95%CI 80.8%–99.3%; Spec. 79.5%, 95%CI 68.4–88.0).

**FIGURE 3:**
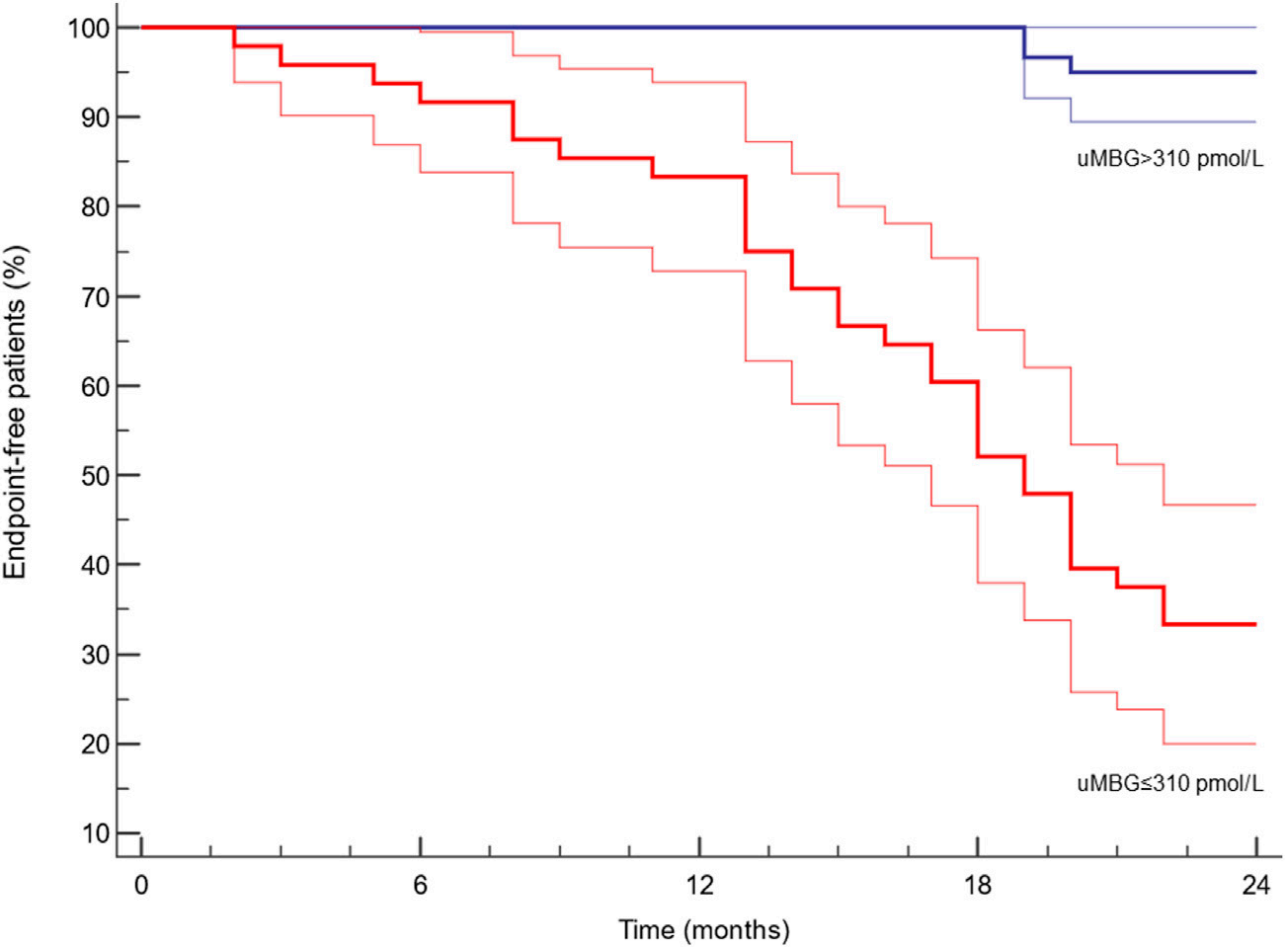
Kaplan-Meier survival curves (95%CIs in blurred lines) of renal endpoint-free individuals after stratification for the ROC-derived cut-off of baseline uMBG levels. Log-Rank test X^2^ = 57.9; p < 0.0001.

ROC analyses revealed a remarkable diagnostic capacity of this biomarker to identify patients with CKD progression with an Area Under the Curve (AUC) of 0.898 [95%CI 0.825–0.948; p < 0.0001, Figure 2) and ≤310 pmol/L as the best cut-off (Youden Index; Sensitivity 94.3%, 95%CI 80.8%–99.3%; Specificity 79.5%, 95%CI 68.4–88.0). Kaplan-Meier survival analyses indicated a significantly faster progression to the renal endpoint in individuals with uMBG≤310 pmol/L (crude HR 13.47; 95%CI 6.62–27.38; p < 0.0001; Log-rank test, χ2 57.9) as compared to others (Figure 3).

### Cox regression analyses for the renal endpoint

Variables that were different at baseline between study subgroups were tested by Cox regression analysis to evaluate the association with the renal outcome. At univariate analyses, 24 h-proteinuria (HR 1.663; 95%CI 1.082–2.432, p = 0.02), eGFR (HR 0.971; 95%CI 0.948–0.994, p = 0.01), age (HR 1.059; 95%CI 1.018–1.103, p = 0.004) and uMBG (HR 0.990; 95%CI 0.987–0.993, p = 0.001) were confirmed as significant predictors of CKD progression in this cohort. Conversely, such an association was not found for BMI, hemoglobin, and systolic blood pressure (p ranging from 0.12 to 0.33). A multivariate Cox model including these univariate predictors reported an independent 9% decrease in the risk of CKD progression per each pmol/L increase in uMBG excretion (HR 0.991; 95%CI 0.987–0.994, p = 0.01). Also, both age (HR 1.012; 95%CI 1.002–1.066, p = 0.04) and a lower baseline eGFR (HR 0.984; 95%CI 0.959–0.992, p = 0.02) remained independently associated with the endpoint while the correlation with 24 h proteinuria was lost. Table 2 summarizes the results from Cox regression analyses.

**TABLE 2:** Univariate and multivariate Cox regression analyses for variables significantly associated with the renal endpoint. Serum creatinine was excluded from analyses to avoid collinearity with eGFR. Statistically significant predictors are highlighted in bold.

	Units of Increase	HR	95% CI	X^2^	p
Univariate analyses					
**uMBG**	**1 pmol/L**	**0.990**	**0.987–0.993**	**14.56**	**0.001**
**Age**	**1 year**	**1.059**	**1.018–1.103**	**8.09**	**0.004**
**eGFR**	**1 mL/min/1.73** **m** ^**2**^	**0.971**	**0.948–0.994**	**5.97**	**0.01**
**24h-Proteinuria**	**1 g/24h/1.73** **m** ^**2**^	**1.663**	**1.082–2.432**	**4.49**	**0.02**
Haemoglobin	1 g/dL	0.767	0.618–1.019	1.93	0.12
BMI	1 kg/m^2^	0.917	0.856–1.006	1.48	0.24
Systolic BP	1 mmHg	1.014	0.819–1.049	0.78	0.33
Multivariate analyses					
**uMBG**	**1 pmol/L**	**0.991**	**0.987–0.994**	**13.5**	**0.01**
**eGFR**	**1 mL/min/1.73** **m** ^**2**^	**0.984**	**0.959–0.992**	**4.97**	**0.02**
**Age**	**1 year**	**1.012**	**1.002–1.066**	**2.02**	**0.04**
24 h-Proteinuria	1 g/24h/1.73 m^2^	1.322	0.882–1.996	0.69	0.22

Legend: BP, blood pressure; BMI, body mass index; eGFR, estimated glomerular filtration rate (CKD-Epi formula); HR, hazard-ratio; uMBG, urinary Marinobufagenin.

### Non-linear association of uMBG with the renal endpoint

Visual inspection of baseline uMBG levels in study subgroups (Figure 1) prompted the exploration of a nonlinear association with the renal endpoint. Indeed, in patients stratified for uMBG quartiles, most cases of CKD progression (65%) occurred in individuals in the first quartile, while the lowest incidence of the renal endpoint was reported in the third quartile. Assuming this latter group as the reference risk category, patients falling within the first and second quartile displayed a remarkably increased risk of endpoint at crude Cox-regression analyses (HR 53.3 [95%CIs 5.84–486.9] and 14.1 [1.69–117], respectively), while a non-significant risk excess was documented among individuals in the fourth quartile (HR 2.34 [0.15–35.8]). Results remained almost unchanged after full adjustment for age, eGFR, and 24 h proteinuria (HR for the first, second, and fourth quartile: 47.5 [4.87–462.6], 13.2 [1.54–113] and 2.50 [0.16–39], respectively; Table 3).

**TABLE 3:** Univariate and multivariate Cox-regression hazard ratios of the composite renal endpoint across tertiles of baseline uMBG excretion and differences in model performance between a linear- and a restricted cubic spline (RCS)-fitting.

Model	uMBG<240 pmol/L	uMBG 240–350 pmol/L	uMBG 350–445 pmol/L	uMBG >445 pmol/L	p of the model
Unadjusted	53.3 [5.84–486.9]	14.1 [1.69–117]	References	2.34 [0.15–35.8]	<0.0001
Fully-adjusted	47.5 [4.87–462.6]	13.2 [1.54–113]		2.50 [0.16–39]	<0.0001

Model	Linear Fitting				RCS fitting				p for non-linearity
	p	X^2^	AIC	BIC	p	X^2^	AIC	BIC	
Unadjusted	<0.0001	39.7	278.4	281.1	<0.0001	62.3	261.7	272.4	<0.0001
Fully-adjusted	<0.0001	42.5	281.5	282.3	<0.0001	62.5	267.6	277.4	0.002

Legend: Fully-adjusted: model adjusting for CKD-Epi eGFR, age and 24 h proteinuria; AIC, akaike information criterion; BIC, bayesian information criterion; uMBG, urinary Marinobufagenin.

The likelihood-ratio test confirmed a better capacity of both the unadjusted and fully adjusted cubic spline models to describe the risk distribution better than the linear counterparts (p < 0.0001 and 0.002, respectively). The gain in model fitting was further supported by the improvement in either the Akaike or the Bayesian criterion (Table 3).

**FIGURE 4:**
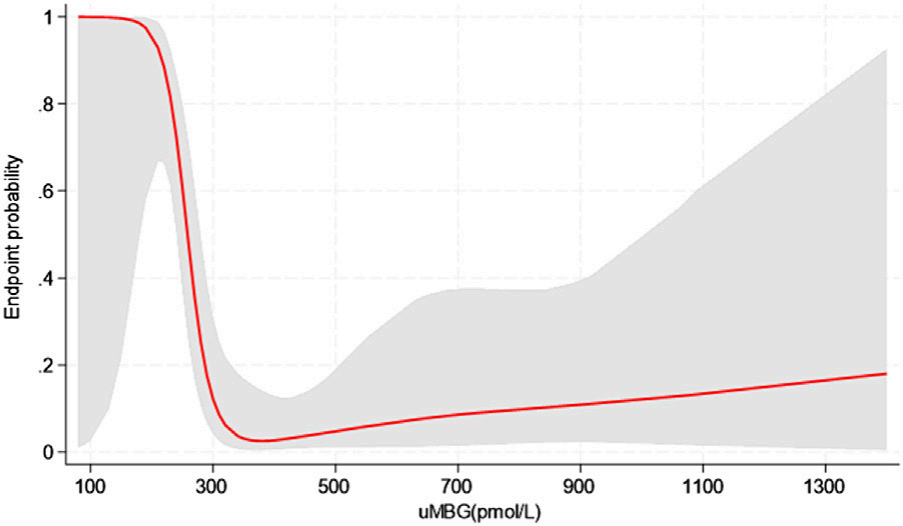
Restricted cubic splines of uMBG displaying the predicted probability of the combined renal endpoint from logistic regression analyses (red line with grey area showing the 95%CIs).

**FIGURE 5:**
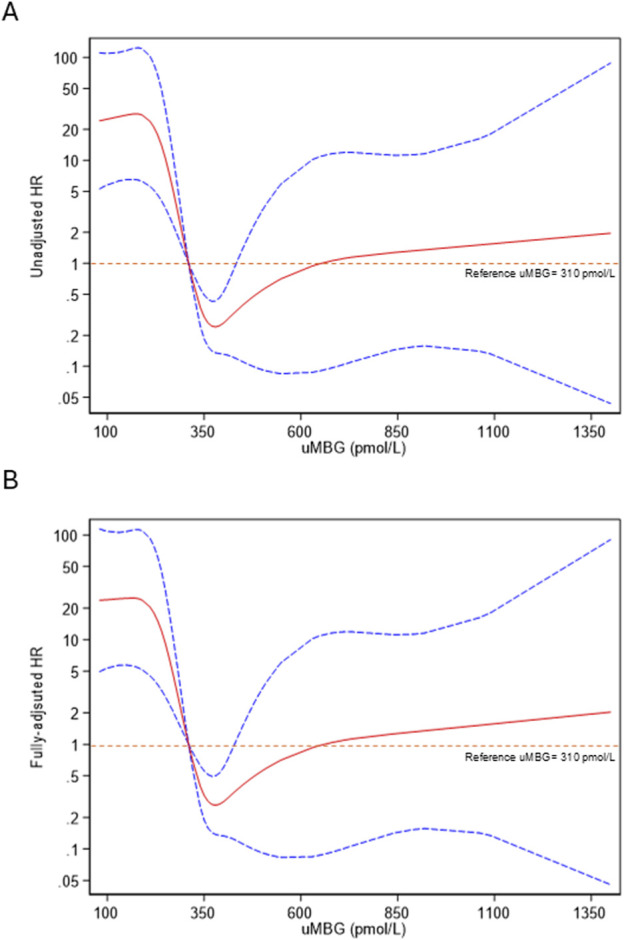
Restricted cubic splines of uMBG fitting **(A)** unadjusted and **(B)** fully adjusted (age, eGFR and 24 h-proteinuria) Cox regression for the combined renal endpoint with the best diagnostic ROC-derived uMBG threshold (310 pmol/L) as the reference risk value. The solid red line represents the HR point estimate, and the dotted lines represent the 95% CIs.

Restricted cubic splines (RCS) of uMBG levels fitting logistic regression analyses demonstrated a reverse J-shaped association between this biomarker and the predicted probability of CKD progression, the highest risk being observed among individuals with the lowest baseline values (Figure 4). By the same token, RCS fitting Cox regression analyses described a reverse J-shaped curve of the estimated HR across uMBG levels, assuming the best diagnostic ROC-derived threshold (310 pmol/L) as the reference risk value. Again, the highest risk probability of CKD progression was observed among individuals with very low uMBG excretion, while a substantial risk plateau was noticed for increasing uMBG above the reference value (Figure 5A). Of note, such a curvilinear pattern remained unchanged after multivariate adjustment for age, eGFR, and 24-proteinuria (Figure 5B) or by considering the median uMBG (350 pmol/L) instead of the best ROC cut-off as the reference risk value.

## Discussion

Whatever its primary cause, CKD progression to ESKD mostly relies on a deranged fibrotic process at the tubulointerstitial level with parenchymal atrophy leading to a consequent, irreversible loss in the overall kidney functionality (Huang et al., 2023).

Solid experimental evidence indicates that MBG can trigger exaggerated collagen synthesis and cellular epithelial-to-mesenchymal transition at the kidney (Fedorova et al., 2009) and the cardiovascular level (Elkareh et al., 2007). This latter effect may explain the causal role of the elevated circulating levels of this hormone in the pathophysiology of uremic cardiomyopathy (Elkareh et al., 2009; Haller et al., 2012), a largely prevalent and dangerous complication of ESKD which is characterized by deranged myocardial fibrosis.

Although mechanistic evidence demonstrating a similar putative effect on CKD progression is lacking, findings from the present study suggest that an altered uMBG excretion may reflect a higher propensity to progressive renal disease in individuals with non-advanced CKD, beyond the information portended by traditional risk markers.

Indeed, in our prospective investigation, patients with CKD progression showed, on average, a baseline uMBG excretion which was almost halved as compared with those maintaining a stable renal function over time. No less important, uMBG was revealed as a fully independent predictor of the renal outcome at Cox-regression analyses while, in this cohort, other generally acknowledged risk factors like 24 h-proteinuria, systolic blood pressure, or BMI (Hannan et al., 2021) were apparently not.

Of note, at multivariate analyses, uMBG ranked as the strongest predictor of CKD progression, with an estimated linear risk increase of about 9% per each 1 pmol/L increase from its baseline levels. Importantly, such a predictive capacity remained unaffected by strong confounders like eGFR and age: this latter observation is of high relevance as it indicates that lower uMBG levels in progressive renal disease could represent more than a simple epiphenomenon of reduced renal clearance, rather suggesting a possible causal role on chronic damage exacerbation.

Beyond a remarkable prognostic capacity, uMBG displayed also a great discriminatory performance at ROC analyses in identifying patients experiencing progressive CKD (AUC 0.898). By the same token, individuals with baseline uMBG values below the optimal ROC-derived cut-off value of 310 pmol/L reported a significantly faster CKD progression at Kaplan-Meier analyses, exhibiting a crude 13.47 HR of worse renal outcomes as compared with those with higher uMBG levels.

Hence, in this population setting, uMBG may serve either as a prognostic or a diagnostic biomarker for progressive renal damage, meaning that this substance could potentially hold usefulness for early detection and screening, as well as for risk stratification, and disease progression monitoring.

Unfortunately, despite being hypothesis-generating, our findings remain confined to a single, homogeneous CKD cohort. The pilot nature and the relatively small sample size of this study cannot rule out the presence of a significant selection bias or residual confounding which may hamper the generalizability of results in CKD populations with more heterogeneous severity or etiology. On top of this, the relatively low number of renal events prevented the possibility of performing more focused analyses on the separate components of the composite endpoint, which further supports the need for future investigations on larger cohorts.

Importantly, although at linear analyses an increase in uMBG excretion was paralleled by a decrease in the risk of CKD progression (‘the higher, the better”), patients falling in the lowest quartile of uMBG displayed a remarkable forty-seven-fold increase in the HR as compared to individuals with intermediate uMBG excretion (3^rd^ quartile-reference group), while such risk remained comparable in those with the highest excretion (>445 pmol/L).

This asymmetric incidence of renal events across quartiles of uMBG suggested that a non-linear trend could better describe the predictive association between this biomarker and the risk of CKD progression.

Indeed, the visual exploration of this association by restricted cubic spline models evidenced a curvilinear, inverse J-shaped trend in either the predicted probability or the HR of CKD progression, with the highest risk excess found for very low uMBG levels below the optimal ROC-derived threshold (310 pmol/L) and a substantial risk plateau noticed in individuals with uMBG above such cut-off. Importantly, this trend was not affected by adjustment for age, eGFR, and 24 h-proteinuria, which gave further proof to the fully independent predictive capacity of this biomarker.

As previously mentioned, the limited study sample and the monocentric nature of the investigation are key limits of our findings. Hence, we cannot verify whether such a particular risk pattern is generalizable to more variegated CKD populations or whether a more extended follow-up observation would somewhat impact this prognostic association. On the other hand, strengths including a rigorous methodological approach, and the establishment of a largely validated and inclusive endpoint with a systematic event collection deserve, in our opinion, to be mentioned as well.

In conclusion, we found very low uMBG excretion to impart relevant prognostic information for disease progression in non-advanced CKD patients with stable renal function. Despite preliminary, our findings may pave the way for future, confirmatory investigations in larger cohorts to ascertain the exact potential of uMBG as a biomarker for improving renal risk stratification in composite CKD populations, beyond traditional risk markers. On top of this, focused mechanistic studies are also desirable to clarify the biological mechanisms linking reduced urinary MBG excretion with chronic renal damage and whether this hormone may represent a therapeutic target for retarding CKD progression.

## Data Availability

The raw data supporting the conclusions of this article will be made available by the authors, without undue reservation.
